# Impaired Inhibitory Control of Saccadic Eye Movements in Cervical Dystonia: An Eye‐Tracking Study

**DOI:** 10.1002/mds.28486

**Published:** 2021-01-08

**Authors:** Federico Carbone, Philipp Ellmerer, Marcel Ritter, Sabine Spielberger, Philipp Mahlknecht, Eva Hametner, Anna Hussl, Anna Hotter, Roberta Granata, Klaus Seppi, Sylvia Boesch, Werner Poewe, Atbin Djamshidian

**Affiliations:** ^1^ Department of Neurology Medical University Innsbruck Innsbruck Austria; ^2^ Interactive Graphics and Simulation Group University of Innsbruck Innsbruck Austria

**Keywords:** eye tracking, cervical dystonia, saccadic inhibition, prefrontal cortex

## Abstract

**Background:**

The pathophysiology of cervical dystonia is still unclear. Recent evidence points toward a network disorder affecting several brain areas. The objective of this study was to assess the saccadic inhibition as a marker of corticostriatal function in cervical dystonia.

**Methods:**

We recruited 31 cervical dystonia patients and 17 matched healthy controls. Subjects performed an overlap prosaccade, an antisaccade, and a countermanding task on an eye tracker to assess automatic visual response and response inhibition.

**Results:**

Cervical dystonia patients made more premature saccades (*P* = 0.041) in the overlap prosaccade task and more directional errors in the antisaccade task (*P* = 0.011) and had a higher rate of failed inhibition in the countermanding task (*P* = 0.001).

**Conclusions:**

The results suggest altered saccadic inhibition in cervical dystonia, possibly as a consequence of dysfunctional corticostriatal networks. Further studies are warranted to confirm whether these abnormalities are affected by the available therapies and whether this type of impairment is found in other focal dystonias. © 2021 The Authors. *Movement Disorders* published by Wiley Periodicals LLC on behalf of International Parkinson and Movement Disorder Society.

Cervical dystonia (CD) is characterized by involuntary activity of cervical muscles leading to involuntary movements and postures of the head, neck, and shoulders.^1^, ^2^ It is often associated with dystonic head tremor and neck pain.^3^ Although CD has traditionally been described as a disorder of basal ganglia motor control; nonmotor symptomns such as depression, obsessive–compulsive disorders, and anxiety are common in this condition.^4^, ^5^ Conflicting results have been published related to the cognitive function of patients with CD, with some studies failing to detect cognitive deficits,^6^, ^7^ others attributing deficits *on cognitive testing to pain and abnormal head movements,* ^8^ *and more recent studies reporting impairment in set shifting and working memory*.^9^ *The latter domains require intact dorsolateral prefrontal cortex (DLPFC) function and a variety of* structural and functional abnormalities of the DLPFC in CD patients have been published.^10^, ^11^ *The DLPFC and its projections via the striatum are important for response inhibition and for* regulating the superior colliculus (SC), a multilayered structure in the midbrain involved in saccadic eye movement generation.^12^ Furthermore, the SC receives projections from other cortical structures such as the frontal eye fields for volitional and the parietal eye fields for reflexive saccades.^13^ Until now, only a few studies have assessed saccadic eye movements in patients with CD, and results have again been inconsistent. Although some studies reported slower saccadic reaction times,^14^ others did not find any difference compared with controls.^15^

Based on findings of DLPFC dysfunction in CD, we hypothesized that CD patients may have difficulties in inhibitory saccadic control compared with healthy volunteers and that abnormalities described in saccadic behavior may help to understand the neural networks involved in this disease.

## 1 Methods

### 1.1 Participants

Forty‐eight subjects were included: 31 patients with isolated or segmental idiopathic CD and 17 age‐ and sex‐matched healthy controls (HCs).

A Mini–Mental State Examination score below 26, psychiatric disorders, or uncorrected visual impairments were exclusion criteria. Drugs affecting the central nervous system were not allowed with the exception of antidepressants, if on a stable dose for 4 weeks prior to testing. CD patients were on regular treatment with botulinum toxin and had received their last botulinum injection at least 90 days prior to testing.^16^

### 1.2 Experimental Protocol

Participants filled out the Barratt Impulsiveness Scale (BIS‐11) and the Hospital Anxiety and Depression Scale. We adopted the Toronto Western Torticollis Rating Scale^17^ and a modified version of the Tsui scale^18^, ^19^ to assess disease and tremor severity in CD patients.

Eye tracking was carried out using a Tobii TX300 system (www.tobii.com). All subjects were tested by the same investigator under identical light conditions in the early afternoon. The assessment consisted of a prosaccade task, an antisaccade task, and a countermanding task, always performed in this order.

(1) In the prosaccade task subjects were required to fixate a target in the middle of the screen; the target disappears, and a peripheral cue appears. Subject had to perform a saccade toward the cue. We employed an overlapping variant, with target and cue on the screen simultaneously for a short time, delaying the visually guided saccade. This task was repeated 80 times. (2) The antisaccade task was cognitively more demanding than a prosaccade: subjects were required to perform a mirror saccade in the opposite direction of the cue. Saccades to cue were considered errors.^20^ This task was divided in 2 blocks of 20 repetitions each.^21^ (3) In the countermanding task the central target was followed by a green arrow anticipating the appearance of the peripheral cue. The arrow was randomly followed by a red stop signal in a fourth of trials. In this case, the subject had to refrain from looking at the peripheral cue. This task was performed 60 times. Anticipatory errors in the prosaccade task, directional errors in the antisaccade task, and inhibition errors in the countermanding task were the main outcome measures.

For each task, reaction times were measured from the appearance of the peripheral cue until the first saccade; any saccade with latency under 50 milliseconds was discarded. In the pro‐ and antisaccade task, reaction times shorter than 140 milliseconds were classified as “express saccades.”^22^ Variance of the reaction times in the prosaccade task were expressed using the coefficient of variation, defined as the interquartile range of the reaction time divided by the median.^23^

Prior to each of the 3 tasks, participants performed a practice run consisting of 4 task repetitions for which verbal feedback was given. A break of a maximum of 2 minutes was allowed between the 3 tasks.

### 1.3 Statistical Analysis

The statistical analysis was carried out using SPSS (v24).^24^ Normality of the data was assessed with Shapiro–Wilk test. Based on the distribution of the data, parametric and nonparametric tests were employed. The level of significance for all analyses was set at a 2‐sided *P* < 0.05.

## 2 Results

### 2.1 Demographics and Disease Characteristics

No differences in sex, age, or education were found. CD patients had higher scores for anxiety and depression symptoms compared with HCs (*P* = 0.010 and *P* = 0.002, respectively). However, none of the cutoff values for depression and anxiety^25^ were reached by subjects in either of the 2 groups. There was no difference in the BIS‐11 total score between the CD and control groups; a subscore comparison revealed a higher score in dystonia patients in the attentional impulsiveness domain (*P* = 0.031).

### 2.2 Saccadic Tasks

**FIG. 1:**
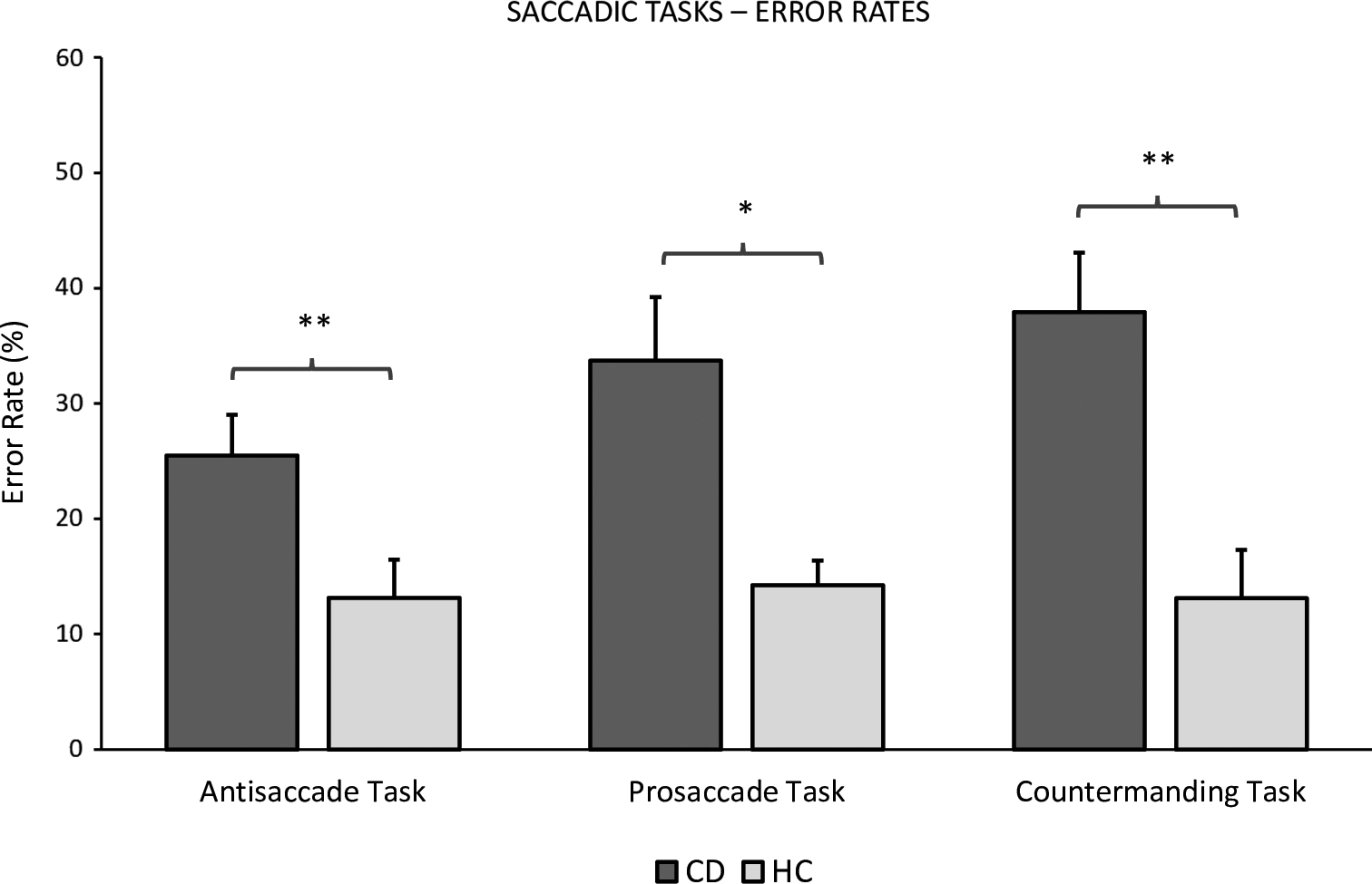
Results and comparison between groups of the saccadic tasks' error rates. Each column represents the mean error rate, each presented with standard error of the mean on top. The directional error is depicted for the antisaccade task, the anticipatory error for the prosaccade task, and the failed inhibition error for the countermanding task. Asterisks represent the difference between groups (*P < 0.05; **P < 0.01). CD, cervical dystonia; HC, healthy controls.

CD patients had higher anticipation errors (*P* = 0.041), made more express saccades (*P* = 0.042) in the pro‐saccade task, had longer reaction times (*P* = 0.036), and made more directional errors at normal and express latencies in the antisaccade task (*P* = 0.011) compared with HCs. Furthermore, patients made more saccades toward the target in the No‐Go trial of the countermanding task (*P* = 0.001). There was no significant difference in reaction time variance between CD and HC (Table 1, Fig. 1).

**TABLE 1:** Results and comparison between groups of the saccadic tasks' error rates and reaction times

Parameters of saccadic tasks	CD			HC			Independent *t* test/Mann–Whitney test
	n	Mean	SD	n	Mean	SD	*P* ^a^
Prosaccade reaction time (ms)	31	269.9	75.6	15	292.9	65.1	0.313
Prosaccade anticipation errors (%)	31	33.7	30.5	15	14.2	8.0	**0.041**
Prosaccadic express saccades (%)	31	21.5	19.3	15	12.5	8.9	**0.036**
Prosaccadic coefficient of variance	31	0.7	0.3	15	0.5	0.1	0.281
Incorrect antisaccade reaction time (ms)	31	210.3	53.1	17	200.9	65.8	0.614
Correct antisaccade reaction time (ms)	31	310.7	72.7	17	259.0	39.2	**0.002**
Antisaccade directional errors (%)	31	25.5	19.7	17	13.1	13.7	**0.011**
Antisaccade express errors (%)	31	20.1	23.7	17	8.6	15.2	**0.039**
Countermanding inhibition errors (%)	31	37.9	28.6	17	13.1	16.7	**0.001**
Countermanding task (Go) Reaction time (ms)	31	209.7	37.3	17	207.8	62.2	0.911
Countermanding task (No‐Go) Reaction time (ms)	31	279.5	88.3	17	298.5	179.8	0.663

^a^ Significant *P* values are represented in bold text.

Abbrevations: CD, cervical dystonia; HC, healthy controls.

Next, we performed a subanalysis on error rates and reaction times in the antisaccade task comparing the first 20 trials (block 1) with the second 20 trials (block 2). Patients made fewer errors (23.0 ± 20.0 vs 27.9 ± 21.1, *P* = 0.02) and had shorter reaction times for correctly performed antisaccades (298.2 ± 70.9 vs 329.3 ± 84.0, *P* < 0.01) in block 2. HCs showed no difference between blocks (*P* > 0.05). Similarly, we analyzed the error rate in the countermanding task dividing it in half. Again patients performed significantly better in the second half (CD, 46.9 ± 31.9 vs 25.9 ± 27.5; *P* < 0.01; HC, 16.4 ± 20.8 vs 8.9 ± 13.6; *P* = 0.09).

To account for possible effects of the laterality of dystonic head rotation, we compared reaction times and error rates separately for either direction (right or left) for every saccadic task. There were, however, no significant differences between saccadic tasks in the direction of the laterality of CD and those in the opposite direction (all *P* > 0.05; see Supplementary Material S1.). There were also no group differences regarding the percentage of hypometric (CD, 5.3 ± 5.9; HC, 6.3 ± 4.0; *P* = 0.512) or hypermetric (CD, 2.0 ± 3.8; HC, 1.9 ± 3.8; *P* = 0.916) saccades in the prosaccade task.

## 3 Discussion

In this study we describe poorer saccadic response inhibition in CD patients compared to HCs. More specifically, CD patients made more anticipatory prosaccades, more directional errors in the antisaccade task, and more errors in the countermanding task.

A loss of inhibition can occur at different levels in patients with focal dystonia.^26^ At least 2 mechanisms of inhibition are required in the antisaccade task: at the beginning of the task a preemptive top‐down inhibition, which relies on intact frontal areas (mainly the DLPFC and frontal eye fields but also the superior colliculus), is necessary to avoid express latency errors. In contrast, once the stimulus appears automated saccades toward the target are suppressed by the supplementary eye field. A failure of this system leads to longer latency errors. Crucially, both these mechanisms are mediated by the basal ganglia. Furthermore, a large network of other brain areas including the thalamus, the cerebellum, the brain stem reticular formation, the parietal eye field, and other cortical areas are necessary for visual fixation and saccadic control.^27^

In this study, CD patients made more directional errors than controls, at both longer and express latencies, implying a dysfunction of both mechanisms. The countermanding task differs from the antisaccade task. Here, the inhibition of an already started action is necessary. In addition to the DLPFC and frontal eye fields, the supplementary eye field and other frontal areas such as the right ventrolateral prefrontal cortex as well as intact basal ganglia function are required.^27^, ^28^

Our results highlight a dysfunction of the frontal cortical top‐down inhibitory control in CD and are also consistent with previous results in other focal dystonias.^29^ In line with this, functional imaging studies have shown that successful top‐down inhibition to prevent the automatic prosaccade relies on an intact network comprising the DLPFC together with the frontal eye field, basal ganglia, and SC.^30^, ^31^ Importantly, imaging studies suggest that this network is altered in CD.^11^ In accordance with our findings, neuropsychological tests have revealed impairment in working memory, cognitive flexibility, and frontal lobe function in patients with CD.^9^, ^32^, ^33^ Finally, disruption of sensory‐motor integration in patients with focal dystonia^34^ may also affect oculomotor performance.^35^

The results of the antisaccade task presented here are in contrast with a previous small study in CD (n = 8).^14^ However, because of the small sample size, a direct comparison of the 2 studies is not possible.

It is important to note that the impairment described here is not specific to CD. Poorer saccadic performance has been previously described in patients with dementia as well as patients with other basal ganglia disorders such as Huntington's disease, atypical parkinsonism, idiopathic Parkinson's disease, and patients with schizophrenia 13, 36, 37, 38, 39strengthening the hypothesis that dysfunction of the corticobasal network, either because of basal ganglia lesions, frontal cortex dysfunction, or both may lead to poorer saccadic control.

We want to highlight a limitation of this study: we used a fixed order for the eye‐tracking paradigms. Future studies should consider using a pseudorandomized order to avoid possible learning effects. Importantly, however, poorer performance of the CD group was not because of fatigue, as patients performed significantly better in the second half of the antisaccade and countermanding task.

In conclusion, we demonstrate impaired saccadic response inhibition in CD patients, which may be because of dysfunction of the corticostriatal network. Saccadic assessment in CD is noninvasive, time, and cost effective and could represent a viable biomarker of disease to be implemented both in research and clinical practice. Further studies are needed to assess whether this impairment is shared by other focal or segmental dystonias.

## Author Roles

(1) Research Project: A. Conception, B. Organization, C. Execution, D. Software design, E. Eye tracking paradigms design; (2) Statistical Analysis: A. Design, B. Execution, C. Review and Critique; (3) Manuscript Preparation: A. Writing of the first draft, B. Review and Critique

F.C.: 1A, 1B, 1C, 1E 2A, 2B, 2C, 3A, 3B.

P.E.: 1E.

M.R.: 1D.

S.S.: 1C.

P.M.: 1C, 3B.

E.H.: 1C.

A.H.: 1C.

A.H.: 1C.

R.G.: 1C.

K.S.: 1A.

W.P.: 2C, 3B.

S.B.: 1B, 1C, 3B.

A.D.: 1A, 1B, 2A, 2C, 3B.

## Supporting information


**Table S1.** Demographic data of study population
