# Supplementary material for: Impaired Inhibitory Control of Saccadic Eye Movements in Cervical Dystonia: An Eye‐Tracking Study
Source: Mov Disord. 2021 Jan 8;36(5):1246–50. doi: 10.1002/mds.28486 (PMC8247854; doi:10.1002/mds.28486)
Supplement: Supplementary file 1 — Table S1. Demographic data of study population [file MDS-36-1246-s001.docx]

**SUPPLEMENTAL MATERIAL**

**Methods**

*Participants*

The study was approved by the Research Ethics Committee of Innsbruck Medical University, Austria, before study initiation.

*Experimental Protocol*

Eye tracking testing was carried out using a Tobii TX300 system (Tobii Technology AB, www.tobii.com), displaying visual stimuli on a 23 inches screen with a 1920x1280 pixels resolution. This video-based eye tracker uses central pupil corneal reflection with dark pupil eye tracking technique. Participants sit comfortably in a quiet and dimly lighted room, keeping a constant distance of 65 cm from the screen. A headrest is used to minimize head movements during testing. Raw eye movement data were acquired with a 300 Hz sampling rate and exported through a dedicated software (Tobii Pro Lab, version 1.83). Before every test a 9-point calibration is performed, in order to obtain a gaze position accuracy under 0.4 degrees.

*Tasks*

1) Pro-saccade task: the target presented is a white cross (dimensions 0.5 x 0.5 degrees) on a black background for a random period of time of either 800ms or 1200ms. Introducing this variability at the beginning of the trial prevents the subject from getting unconsciously used to the test structure and possibly increasing the number of premature automated saccades to cue in the following part. The cue is a white point (radius 0.5 degrees), appearing 10 degrees to the right or to the left of the cross. The overlapping phase lasts randomly either 300ms or 700 ms, afterwards the cue stays on the screen 1000 ms. This task is repeated 80 times in one block.

2) Anti-saccade task: the same target and cue used in the pro-saccade task are employed. The central target stays on the screen for 1500 ms and is then simultaneously replaced by the peripheral cue, appearing randomly 10 degrees to the right or to the left for 1000 ms.

3) Countermanding task: the same target and cue as in the other two tasks are used. The central target appears on the screen for 1100 ms, followed by a green arrow for 100ms pointing right or left, anticipating where the peripheral cue will appear and therefore in which direction the saccade will have to be performed. The cues appear always 10 degrees to the right or to the left of the target, in a random order. In some trials however, the arrow is followed after 100 ms by a red stop signal. In this case, the subject must refrain from looking at the peripheral cue and instead keep his gaze where the central target was. This task is performed in one block with 60 repetition, with the stop signal randomly present in 20 trials.

*Analysis*

The raw data are exported as TSV files and processed with a light-weight standalone software written in F# and designed specifically for these assessments. The analysis algorithm uses the data provided by the Tobii scanner and does an on-the-fly data stream analysis. The source code for the software is available on Github.^1^ The data are visualized as angle error for each time point, using as a reference a fix point defined in Tobii Pro Lab. The eye movements are classified as fixations or positive and negative angle deviations. A linear regression is used to elaborate this data. Based on the classification and the regression the software defines automatically the reaction times of the participants. Every single trial is manually controlled and revised; the rater can additionally correct the reaction times and mark each trial based on data quality and performance of the participant (i.e. define hypometric/hypermetric saccades, early initiated saccades, bad quality trials). The final data is then exported as an excel file, ready for the subsequent analysis.

**Results**

The demographical data and disease characteristics are summarized in table 2.

*Saccadic tasks*

A data quality threshold of 75% was set for every task. Two healthy controls did not reach this threshold in the pro-saccade task (data quality: 14.8%, 24.0%) and those trials were accordingly not included in the statistical analysis. The mean data quality for pro-saccade, anti-saccade, and countermanding tasks was 92.7%, 96.1%, and 92.5% respectively.

Based on the duration of the overlapping period in this task (300ms or 700ms) the percentage of the anticipation error varied similarly in both groups: a shorter overlapping window was associated with a lower error rate (CD 22.5±25.7%, HC 8.4±8.4%) and conversely longer overlapping times were associated with a higher error rate (CD 33.1±27.8%, HC 10.5±9.1%). A Wilcoxon-signed ranked test showed that the increase in error rate was significant in the CD group (Z= -3.856, p<0.001), but not in healthy controls (Z= -0.659, p= 0.511). The between-group comparison was repeated for both overlapping windows. A Mann-Whitney test confirmed that the anticipatory error rate was significantly greater for CD patients in the 700ms overlapping period (p= 0.001), while in the 300ms trials it was almost significant (p=0.053).

A Spearman´s rank-order correlation was run to determine the relationship between saccadic performance and disease characteristics. No linear correlation was found between errors in the three saccadic tasks and disease severity (defined by total modified Tsui score, total TWSTRS, or any subscale of the TWSTRS; all p values>0.05). A significant linear correlation was found between performances in the three tasks

A Spearman’s rank-order correlation showed a significant correlation between anticipatory errors in the pro-saccade task and directional errors in the anti-saccade task (r_s_=0.349, p=0.019), a correlation between anticipatory errors in the pro-saccade task with failed inhibition errors in the countermanding task (r_s_= 0.525, p<0.001) and a correlation between directional errors in the anti-saccade task and failed inhibition errors in the countermanding task (r_s_=0.597, p<0.001)

CD patients had lower rate of express saccades towards the side of torticollis rotation (16.7±3.4) compared to the opposite direction (19.5±3.4). However, this difference was not significant (p=0.598). Furthermore, there were no significant differences in the direction of the laterality of CD compared to the opposite direction in pro-saccade performance (p=0.613), anti-saccade performance (p=0.533), and countermanding task performance (p=0.499).

**References**

1. https://github.com/gileoo/EyeTrackingFS.

| **Table 2**. Demographic data of study population | | | | | |
| --- | --- | --- | --- | --- | --- |
|  | **CD Patients (n=31)** | | **Controls (n=17)** | | **P value** |
| Sex F (%) | 74.2 |  | 64.7 |  | 0.500 |
| Handedness R (%) | 93.6 |  | 76.5 |  | 0.151 |
| Age | 61.3 | ± 9.8 | 55.8 | ± 11.4 | 0.094 |
| MMSE^a^ | 28.9 | ± 1.2 | 29.1 | ± 1.0 | 0.586 |
| Education (years) | 11.7 | ± 2.6 | 11.7 | ± 1.8 | 0.896 |
| HADS-A^b^ | 5.3 | ± 2.3 | 3.4 | ± 2.3 | **0.010** |
| HADS-D^b^ | 3.9 | ± 2.8 | 1.7 | ± 1.8 | **0.002** |
| BIS-11^c^ | 62.1 | ± 6.5 | 59.1 | ± 6.5 | 0.194 |
| BIS-11 Attentional | 15.9 | ± 2.9 | 13.9 | ± 2.8 | **0.031** |
| BIS-11 Motor | 21.4 | ± 3.1 | 21.5 | ± 3.1 | 0.903 |
| BIS-11 Nonplanning | 24.8 | ± 3.5 | 24.1 | ± 3.1 | 0.467 |
|  |  |  |  |  |  |
| Disease duration (years) | 17.3 | ± 8.9 |  |  |  |
| Time since botulinum injection (days) | 100.4 | ± 11.7 |  |  |  |
| Tsui Score | 4.7 | ± 2.8 |  |  |  |
| Disease Severity (TWSTRS^d^) | 15.3 | ± 5.0 |  |  |  |

^a^Mini Mental State Examination; ^b^Hospital Anxiety Depression Scale; ^c^Barratt Impulsiveness Scale; ^d^Toronto Western Spasmodic Torticollis Rating Scale;
